# Serum trefoil factor 3 is a promising non-invasive biomarker for gastric cancer screening: A monocentric cohort study in China

**DOI:** 10.1186/1471-230X-14-74

**Published:** 2014-04-11

**Authors:** Zhigang Huang, Xie Zhang, Hongna Lu, Lina Wu, Danping Wang, Qiubo Zhang, Huaxin Ding

**Affiliations:** 1 Department of Gastroenterology, Lihuili Hospital of Ningbo Medical Center, 57# Xingning Road, Ningbo 315000, China; 2 Ningbo Diagnostic Pathology Center, 2# Guanghua Road, Ningbo 315000, China; 3 Ningbo University, 818# Fenghua Road, Jiangbei District, Ningbo 315000, China

**Keywords:** Gastric cancer, Diagnosis, Serum trefoil factors, Pepsinogen

## Abstract

**Background:**

The search for better non-invasive biomarkers for gastric cancer remains ongoing. We investigated the predictive power of serum trefoil factor (TFF) levels as biomarkers for gastric cancer in comparison with the pepsinogen (PG) test.

**Methods:**

Patients with gastric cancer, chronic atrophic gastritis (CAG) or chronic non-atrophic gastritis (CNAG), and healthy people were recruited. Serum concentrations of TFFs, PG I, and PG II, as well as the presence of antibodies against *Helicobacter pylori*, were measured by enzyme-linked immunosorbent assays (ELISA). Receiver operating characteristics (ROC) were used to compare the predictive powers of the selected factors.

**Results:**

The serum concentrations of TFF1, TFF2, and TFF3 in the control groups were significantly lower than those in the gastric cancer group with the exception of TFF2 which was elevated in CAG. The area under the ROC curve for TFF3 was greater than that for the PG I/II ratio (0.81 vs 0.78). TFF3 also had a significantly higher predictive power for distinguishing gastric cancer than the PG test (odds ratio: 10.33 vs 2.57). Moreover, combining the serum TFF3 and PG tests for gastric cancer had better predictive power than either alone.

**Conclusions:**

Serum TFF3 may be a better predictor of gastric cancer than the PG test, while the combined testing of serum PG and TFF3 could further improve the efficacy of gastric cancer screening.

## Background

Gastric cancer is the second most frequent cause of cancer death. Approximately one million new cases of gastric cancer are diagnosed annually worldwide [1]. In most countries, including China, gastric cancer is usually detected at an advanced stage when the prognosis is poor. In Japan, an extensive screening program using photofluorography and endoscopy has succeeded in diagnosing the majority of gastric cancers at earlier stages, which has led to a 40–60% decrease in associated mortality [2-4]. However, widespread endoscopy screening is currently unavailable in China. Therefore, the pre-selection of high-risk individuals with a simple and efficacious non-invasive biomarker, prior to endoscopic examination, has been proposed as a reasonable strategy for gastric cancer mass screening.

Serum pepsinogen (PG) testing, a current method for gastric cancer screening, has the advantage of being simple and inexpensive. It has been used as part of large-scale screening in Japan. However, because of insufficient evidence, the PG test has not yet been recommended for population-based screening [5,6]. Moreover, while a combination of serum PG and *Helicobacter pylori* (HP) antibody testing has been reported to be superior to PG testing alone for predicting gastric cancer risk, this method has also not yet reached a level where it can be directly used to screen for gastric cancer [7].

The trefoil factor (TFF) family consists of three thermostable and protease-resistant proteins, TFF1, TFF2, and TFF3 [8]. These proteins are thought to play a pivotal role in mucosal protection against damage [9]. Their oncogenic potential has also been reported to be associated with cell proliferation, apoptosis, migration, and invasion and angiogenesis [10-16]. The expression of these peptides in the gastrointestinal tract occurs in a tissue- and cell-specific manner. TFF1 and TFF2 are predominantly expressed in gastric mucosa [17], while TFF3 is expressed in goblet cells of the intestine and also at lower levels in other organs such as the breast, salivary gland, respiratory tract, and hypothalamus [18-22]. Recently, serum levels of TFFs in cancer patients, including those with gastric cancer, have been reported to be increased and therefore could be useful biomarkers for screening [23-27].

In our study, we investigated the serum levels of TFFs in patients with gastric cancer. The efficacy of serum levels of TFFs as biomarkers of gastric cancer was further analyzed in comparison to PG testing.

## Methods

### Subjects

Seventy-two patients with gastric cancer who underwent treatment from January 2012 to October 2012 at the Department of Gastrointestinal Surgery at the Ningbo Medical Center of Lihuili Hospital were recruited for this study. Serum samples were obtained before treatment. Clinicopathological data including the TNM stage of tumors and the histological type, according to Lauren classification, were also collected. Sixty-one patients with chronic atrophic gastritis (CAG) and 27 patients with chronic non-atrophic gastritis (CNAG) were also recruited from the Department of Gastrointestinal Endoscopy from March 2012 to July 2012. CAG and CNAG were diagnosed by endoscopic pathohistology, where CAG was defined as a loss of gastric glandular cells or their replacement by intestinal and fibrous tissue in the antrum or corpus of the fundus by biopsy. The serum samples of 37 healthy people, who reported no history of upper gastrointestinal disorders, were obtained from the Health Check Center of Lihuili Hospital from July 2012 to October 2012. Subjects were excluded if they presented with severe comorbidities including hepatic, renal, cardiopulmonary, and hematologic disease, or had previously undergone upper gastrointestinal surgery or vagotomy. Written informed consent was obtained from all participants in accordance with the Declaration of Helsinki. Approval from the research ethics committee of Lihuili Hospital was also obtained.

### Immunoassays for TFFs, Pepsinogen I, Pepsinogen II, and Anti-HP IgG

Serum collected from fasted patients with gastric cancer, CAG and CNAG, or the healthy controls were stored at -80°C until analysis. Serum TFFs, pepsinogen I, pepsinogen II, and anti-HP immunoglobulin (Ig) G levels were measured by enzyme-linked immunosorbent assays (ELISA).

Specifically, serum levels of TFF1, TFF2, and TFF3 were measured using commercial ELISA kits purchased from USCN Life Science (Wuhan, China) and performed according to the manufacturer’s instructions. Briefly, purified polyclonal antibodies were coated onto a 96-well microtiter plates. Next, 100 μl of assay buffer, as a negative control, serum samples, or dilutions of the appropriate human TFF standard, were added to their respective wells and the plates were incubated for 2 h at 37°C. The plates were then washed and the appropriate diluted biotin-labeled TFF polyclonal antibody was added to each well. After incubation for 1 h at 37°C, the plates were washed and diluted streptavidin conjugated to horseradish peroxidase was added to each well. Following incubation for a further 30 min at 37°C, the plates were washed and tetramethyl benzidine (TMB) solution was added for 20 min at 37°C. Finally, stop solution was added to each well, and the absorbance at 450 nm was measured. The concentrations of human TFFs in the samples were then calculated from the working standard curve. The assay sensitivities for TFF1, TFF2, and TFF3 were 44, 13.5 and 52 pg/ml, respectively.

The serum concentrations of PG I and PG II were measured by chemiluminescent enzyme immunoassay kits from Biohit Plc (Helsinki, Finland). The serum PG status was considered positive (PG +) for predicting gastric cancer when the serum PG I level was ≤ 70 ng/mL and the PG I/II ratio was ≤ 3.

HP infection was diagnosed by the detection of serum HP IgG antibody using a commercial enzyme immunoassay kit (Biohit Plc).

### Statistical analysis

All statistical analysis was performed using Graphpad Prism 5.01 (La Jolla, CA, USA). Continuous data of patients and controls were firstly checked to confirm whether they were close to a normal distribution, and then statistically analyzed by t-test for normal distributions or by the Mann—Whitney test for non-normal distributions. A two-sided P value of <0.05 was considered statistically significant. The receiver operating characteristic (ROC) curves and the area under these curves (AUC) were calculated to compare the predictive powers of selected factors.

## Results

### Baseline characteristics of patients and controls

The baseline characteristics of the study subjects are shown in Table 1. The average age of the 72 patients with gastric cancer was 61.7 ± 1.4 years (male/female ratio = 1.23), and that of the 61 CAG—patients was 56.7 ± 1.4 years (male/female ratio = 0.91), thus there was a 5-year age difference between the two groups. The average age of CNAG patients was 48.1 ± 2.8 years, and that of healthy subjects was 56.7 ± 2.8 years, thus the CNAG group was 8.6 years younger than the healthy group.

**Table 1:** The baseline characteristics and HP infection status, serum PG test of gastric cancer and controls

	**Gastric cancer (n = 72)**	**CAG (n = 61)**	**CNAG (n = 27)**	**Healthy control (n = 37)**
**Age(years, mean ± SD)**	61.7±1.4ζ	56.7±1.4	48.1±2.8	56.7±2.8
**Male/Female ratio**	1.23	0.91	0.5	1.31
**Histological type**				
Intestinal type	25(34.7%)			
Diffuse type	47(65.3%)			
**TNM stage**				
Early gastric cancer	16(22.2%)			
Advanced gastric cancer	56(77.8%)			
**HP infection status**				
HP positive	48(66.7%)	30(49.2%)	14(51.8%)	18(48%)
**PG test**				
PG I (ng/ml)	76.91±4.78*	72.02±5.48	79.64±5.74	84.52±4.44
PG II (ng/ml)	25.10±2.35**	19.44±1.27	18.27±1.93	14.39±1.12
PG I/II ratio	3.91±0.29ξ	3.99±0.24	5.84±0.72	7.18±0.69

ζ: No difference between gastric cancer and healthy group (*P* = 0.08); gastric cancer vs CAG P = 0.0179; gastric cancer vs CNAG, P < 0.001.

*: No difference between cancer group and CAG, CNAG and healthy group.

**: Gastric cancer vs. CAG, *P* = 0.0459; gastric cancer vs. CNAG, *P* = 0.0927; Gastric cancer vs. healthy, *P* = 0.002.

ξ: Gastric cancer vs. CAG, *P* = 0.8485; gastric cancer vs. CNAG, *P* = 0.0038; gastric cancer vs. healthy, *P* < 0.0001.

In the gastric cancer group, 16 (22.2%) patients had early stage gastric cancer (stage 0, IA and IB), while 56 (77.8%) patients had advanced gastric cancer. According to Lauren classification, 47 (65.3%) patients presented with diffuse type gastric cancer, while the other 25 (34.7%) showed intestinal type. The HP infection status was very close with a HP + range of 48—66.7% across groups. Serum levels of PG I were also not significantly different between patients with gastric cancer and healthy controls. Serum levels of PG II, however, were significantly higher in the gastric cancer group than those in the healthy group. The PG I/II ratio in the gastric cancer group was significantly lower than that for controls with the exception of the CAG cases (Table 1).

### Serum concentrations of TFFs

**Figure 1:**
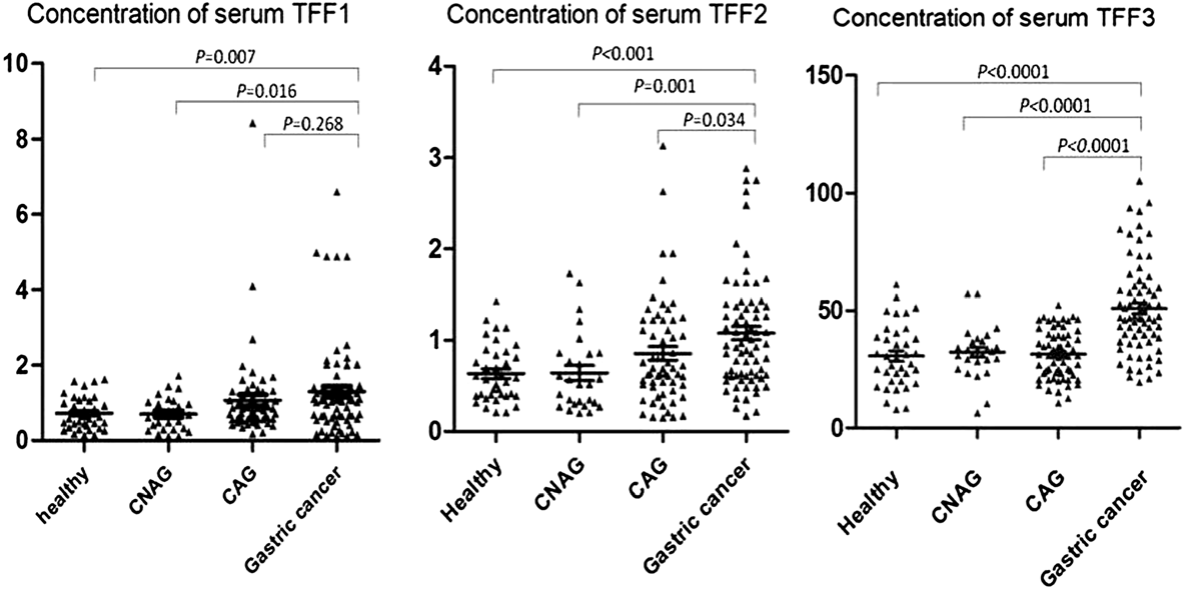
**Concentration of serum TFFs measured by ELISA.** Concentration of serum TFFs in patients with gastric cancer were significantly higher than the other control groups.

The serum concentrations of TFF1, TFF2, and TFF3, in patients with gastric cancer, CAG and CNAG, and in the healthy groups, are shown in Figure 1. In patients with gastric cancer, the mean serum TFF1 concentration was 1.30 ± 0.15 ng/ml (95% CI [1.01, 1.59]), while in CAG, CNAG, and the healthy group it was 1.07 ± 0.14 ng/ml (95% CI [0.77, 1.35]), 0.70 ± 0.08 ng/ml (95% CI [0.53, 0.87]), and 0.72 ± 0.07 ng/ml (95% CI [0.58, 0.86]), respectively. Further statistical analysis revealed that the mean serum TFF1 level in gastric cancer was significantly higher than those in both CNAG (*P* = 0.0075) and healthy group (*P* = 0.0045) patients, however, it was not significantly different from that in CAG (*P* = 0.1332). The mean serum TFF2 concentration in patients with gastric cancer was 1.08 ± 0.07 ng/ml (95% CI [0.93, 1.23]), which was significantly higher than those in CAG (0.86 ± 0.07 ng/ml, 95% CI [0.71, 1.00], *P* = 0.034), CNAG (0.64 ± 0.08 ng/ml, 95% CI [0.47, 0.81], *P* = 0.0011), and healthy (0.63 ± 0.05 ng/ml, 95% CI [0.53, 0.74], *P* < 0.0001) group. The mean TFF3 serum level in gastric cancer patients was also significantly higher than those in the other groups. Indeed, the mean serum TFF3 concentration in patients with gastric cancer was 50.95 ± 2.31 ng/ml (95% CI [46.35, 55.55]), while in CAG, CNAG and healthy groups it was 31.41 ± 1.34 ng/ml (95% CI [28.74, 34.09], *P* < 0.0001), 32.30 ± 2.09 ng/ml (95% CI [28.00, 36.59], *P* < 0.0001) and 30.67 ± 2.20 ng/ml (95% CI [26.22, 35.13], *P* < 0.0001), respectively.

### ROC analysis of serum TFF and the PG test as indicators of gastric cancer

**Figure 2:**
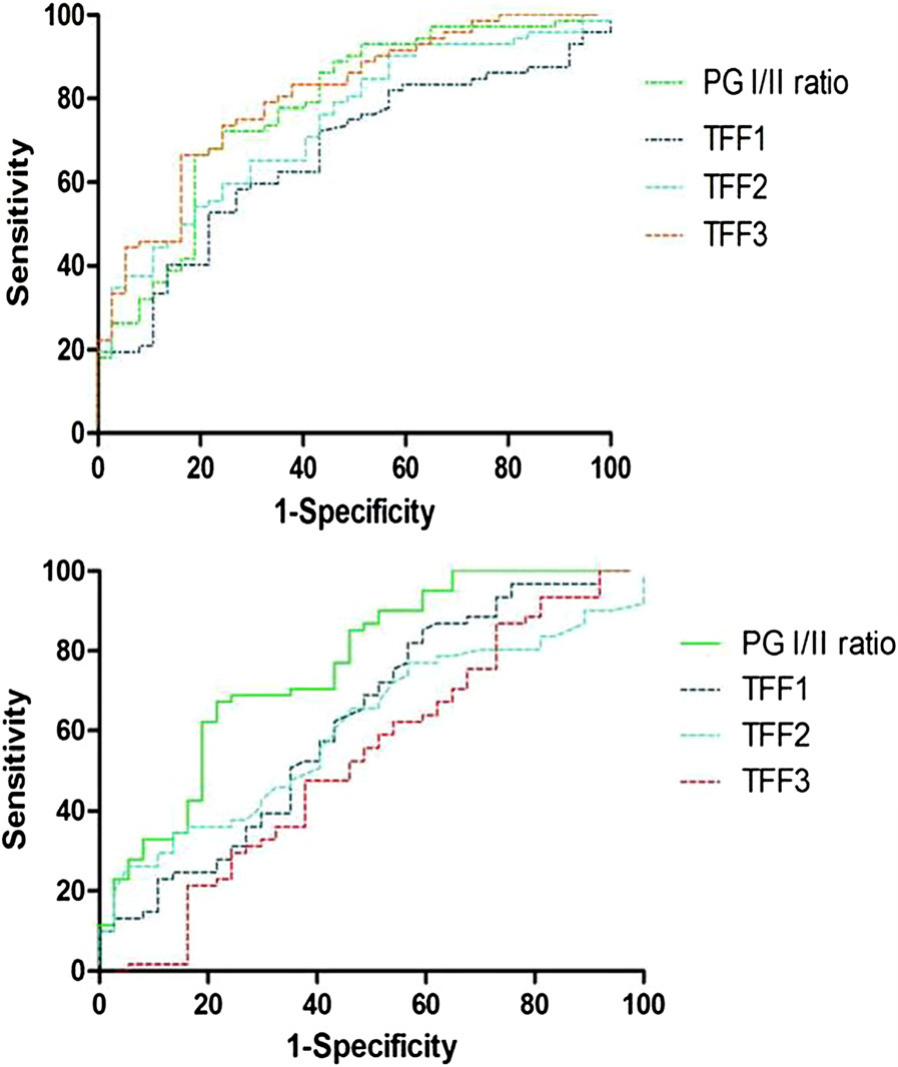
**ROC curves for concentration of serum TFFs and PG I/II ratio to diagnose gastric cancer or CAG. (A)** ROC curves for concentration of serum TFFs to diagnose gastric cancer compared with PG I/II ratio. The area under the curve of serum TFF1, TFF2, TFF3 and PG I/II ratio was 0.67, 0.74, 0.81 and 0.78, respectively. The results showed that serum TFF3 has a greater predictive power for gastric cancer than PG I/II ratio. **(B)** ROC curves for concentration of serum TFFs and PG I/II ratio to diagnosis CAG. The area under the curve of TFF1, TFF2, TFF3 and PG I/II ratio was 0.63, 0.61, 0.53 and 0.76, respectively. The results presented that PG I/II ratio is an obviously better marker for CAG detection than all of serum TFFs.

ROC analysis was performed to evaluate the accuracy of serum concentrations of TFFs and the PG I/II ratio for the diagnosis of gastric cancer. The area under the curve for TFF1, TFF2, TFF3, and the PG I/II ratio were 0.67 (95% CI [0.56, 0.77]), 0.74 (95% CI [0.65, 0.83]), 0.81 (95% CI [0.72, 0.89]) and 0.78 (95% CI [0.69, 0.87]), respectively (Figure 2A). Thus ROC curves indicated a higher observed accuracy for TFF3 when compared with that for the PG I/II ratio. In contrast, for CAG, the area under the curve for TFFs showed significantly lower values when compared with that for the PG I/II ratio (Figure 2B).

The cutoff values for TFF1, TFF2, and TFF3, as calculated by ROC, were 1.0 ng/ml, 0.7 ng/ml, and 42.0 ng/ml, respectively. The sensitivity and specificity of TFF1 were 58.33% and 72.97%, respectively, and the odds ratio was 3.78. The sensitivity and specificity of TFF2 were 65.28% and 70.27%, respectively, and the odds ratio was 4.44. The sensitivity and specificity of TFF3 were 66.67% and 83.78%, respectively, the odds ratio was 10.33. The sensitivity and specificity of PG + were 37.5% and 81.08%, respectively, and the odds ratio was 2.57. These data suggest that the serum concentrations of TFFs, especially TFF3, are significantly associated with gastric cancer as demonstrated by the significantly higher odds ratios than that determined for the PG test (Table 2).

**Table 2:** Comparison of sensitivity and specificity of serum TFFs and PG test for gastric cancer

**Criteria**	**Sensitivity**	**Specificity**	**Odds ratio**
PG test (+)	37.50%	81.08%	2.57
TFF3 (≥42ng/ml)	66.67%	83.78%	10.33
TFF2 (≥0.7 ng/ml)	65.28%	70.27%	4.44
TFF1 (≥1.0 ng/ml)	58.33%	72.97%	3.78

### Effect of HP infection on the ROCs of serum TFFs

**Figure 3:**
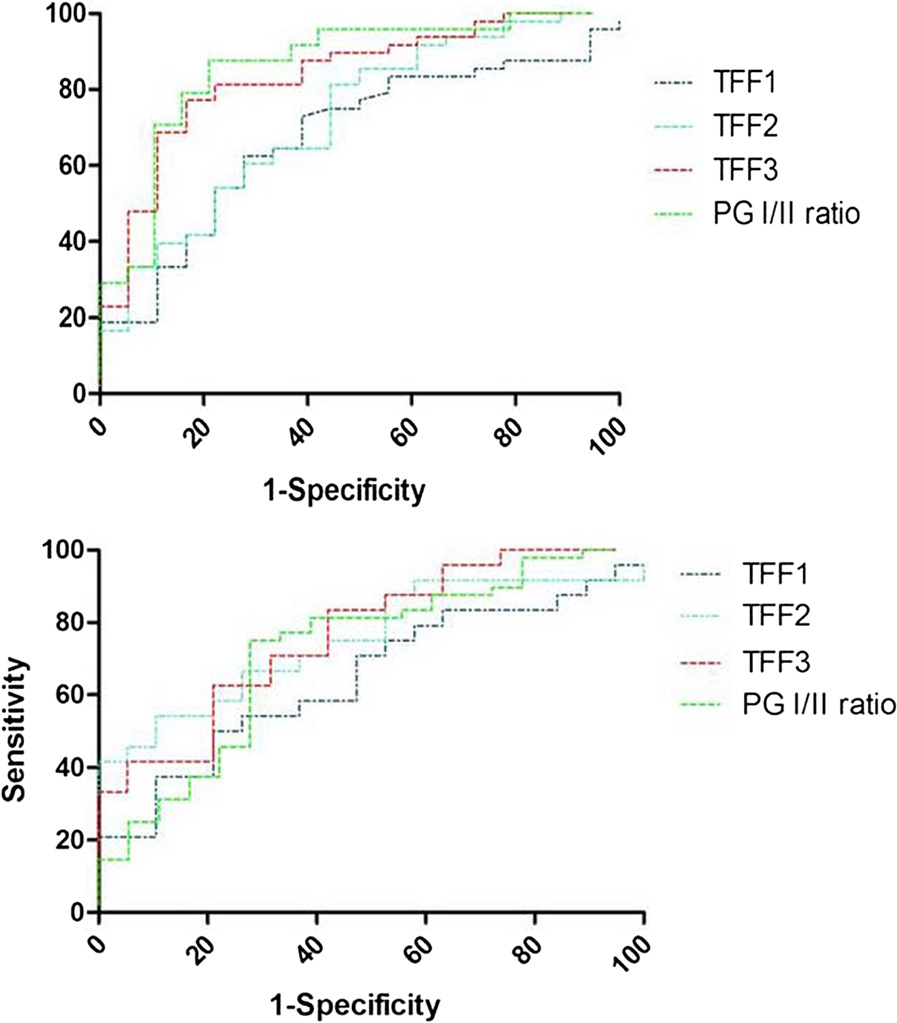
**For HP+/- patients, ROC curves of serum TFFs and PG I/II ratio. (A)** For HP positive patients, the area under the curve for serum TFF1, TFF2, TFF3 and PG I/II ratio was 0.67, 0.72, 0.83 and 0.86, respectively. Serum TFF3 and PG I/II ratio showed a good ROC curve. **(B)** For HP negative patients, the area under the curve for serum TFF1, TFF2, TFF3 and PG I/II ratio was 0.64, 0.75, 0.77 and 0.72, respectively.

To further evaluate the predictive power of TFFs and PG I/II, the gastric cancer and healthy groups were further subdivided according to HP positivity and then ROC analysis was performed. The AUC for HP positive gastric cancer patients were significantly larger for TFF3 (0.83, 95% CI [0.73, 0.94]) and PG I/II ratio (0.86, 95% CI [0.74, 0.98]) than those for either TFF1 or TFF2 (Figure 3A). In contrast, the AUC for HP negative group was slightly smaller than that of HP positive. The AUC for TFF3, TFF2, and PG I/II ratio were very close at 0.77, 0.75 and 0.72, respectively (Figure 3B). These results indicate that TFF3 is a slightly better marker than PG I/II ratio for detecting gastric cancer irrespective of the HP infection status.

### Effect of combining measurement of serum TFF3 and the PG test for gastric cancer determination

We next analyzed the accuracy of using both the concentration of TFF3 and the PG test for detection of gastric cancer. According to the criteria of PG +, 24 of the 72 gastric patients were detected by the PG test. However, when the serum TFF3 test was added, 54 of the 72 gastric cancer patients with gastric cancer were detected, that is, an additional 30 patients with gastric cancer who were not identified by the PG test alone were picked up by the serum TFF3 test. On the contrary, 6 patients with gastric cancer who were not detected by serum TFF3 were positive by the PG test. Thus although the sensitivity of the combined tests increased to 75%, the specificity was decreased.

### The relationships between serum TFFs and the histological types and TNM stages of gastric cancer

**Figure 4:**
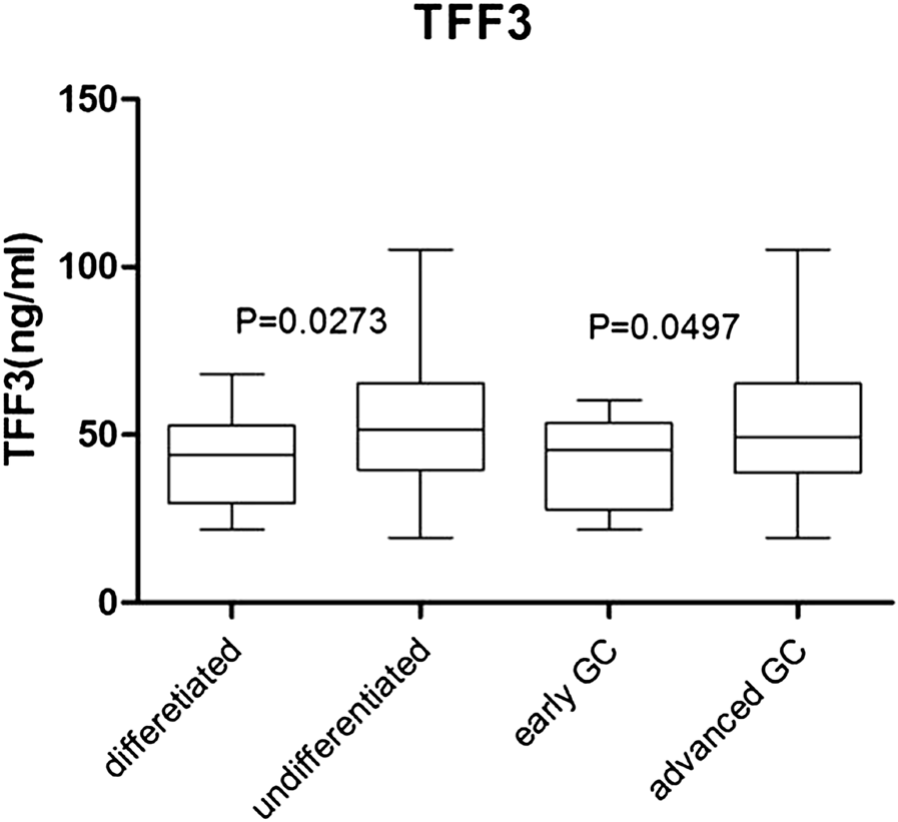
**Distribution of serum TFF3 in differentiated or undifferentiated, early or advanced gastric cancer.** The concentration of serum TFF3 in patients with differentiated gastric cancer was significantly lower than in undifferentiated group (*P* = 0.0273). Serum TFF3 level in patients with early gastric cancer was also significantly lower than in advanced gastric cancer (*P* = 0.0497). The results showed that the concentration of serum TFF3 has correlated with histological type and TNM stages of gastric cancer.

The concentrations of serum TFFs were compared with histological types and TNM stages of gastric cancer to examine their influence on gastric cancer development and progression. The concentration of serum TFF1 did not significantly differ among different histological types or TNM stages. The concentration of serum TFF2, was significantly lower in patients with intestinal type than diffuse type gastric cancer (0.87 ± 0.07 vs 1.19 ± 0.10, *P* = 0.0373), but it was not different between early and advanced stages of gastric cancer. The concentration of serum TFF3 in patients with intestinal type gastric cancer was significantly lower than that in diffuse type (43.87 ± 2.74 vs 54.72 ± 3.10, *P* = 0.0242), it was also reduced in patients with early gastric cancer than in those advanced gastric cancer (42.50 ± 3.32 vs 53.36 ± 2.74, *P* = 0.0497) (Figure 4). As a control, PG I/II ratios were not significantly different in either different histological types or TNM stages of gastric cancer.

## Discussion

Gastric cancer is one of the most common malignancies. It is usually detected at an advanced stage where prognosis is poor and the survival rate is low. To reduce disease-related mortality and improve survival, better biomarkers are needed for the screening and early detection of gastric cancer. The pepsinogen test has been used for gastric cancer screening in Japan [28,29] and has recently started to be used in China. The sensitivity of the pepsinogen test has been reported to range from 45—77% with specificity ranging from 68—87% [30-32]. In the present study, the sensitivity of the pepsinogen test was 37.5%, while its specificity was 81.1%, and the odds ratio was 2.57. The relatively lower sensitivity of the pepsinogen test in our study, when compared with the reported range, may be associated with the use of different PG immunoassay kits. Iijima K et al. [33] have previously reported that serum PG I levels determined using the GastroPenal test (Biohit Plc) were twice as high as those detected with the Japanese kit, although PG assays with both kits were able to identify highly significant correlations between PG concentration and gastric cancer. Additionally, our results may be impacted by the higher rate of diffuse type gastric cancers in our study recruits. Indeed, serum PG II levels have been reported to be increased in patients with diffuse type cancer [34]. In short, it seems that the PG test for gastric cancer is easily influenced by various factors and therefore does not meet the ideal criteria for screening.

Kaise M et al. [35] were the first to report that serum levels of TFFs, especially TFF3, are significantly linked to the presence of gastric cancer. In our cohort, serum concentrations of TFFs showed significantly higher odds ratios than the pepsinogen test. Of the three TFFs, the best biomarker was serum TFF3 which had a sensitivity of 66.67%, a specificity of 83.78%, and an odds ratio of 10.33.

The comparative analysis of the pepsinogen test and serum TFF concentrations for the screening of gastric cancer further shed light on their respective predictive power. We found that 48 (66.7%) of the 72 patients with gastric cancer were negative for the pepsinogen test, while the serum TFF3 test identified an additional 30, the sensitivity of combining the results of serum TFF3 and pepsinogen tests was 75%, which was better than that of either tests alone.

In the HP positive subjects, the AUC of serum TFF3 was very close to that of the PG I/II ratio (0.83 vs. 0.86). In the HP negative subjects, however, the AUC of serum TFF3 was slightly larger than that for the PG I/II ratio (0.77 vs. 0.72). The serum TFFs and PG tests are both based on histological changes in the gastric mucosa from atrophic gastritis. In this study, HP infection was determined by measuring serum anti-HP IgG levels. However, anti-HP IgG levels are reported to decrease when atrophic gastritis has extended to most of the fundic area of the stomach after long-term HP infection [36]. Thus, HP positive subjects in this study may include patients with gastric cancer and non-cancer individuals with the same extent of severity of atrophic gastritis. Subsequently, it is difficult to screen for gastric cancer by using atrophic gastritis related markers in this context. Indeed,the predictive power of the HP infection status was less than that of either serum TFF3 or the PG test.

We also evaluated the relationship between TFF3 and the histological type and stage in gastric cancer. We found that serum concentrations of TFF2 and TFF3 in patients with intestinal type gastric cancer were lower than those in patients with diffuse type. Neither serum TFF1 or the PG I/II ratio were significantly associated with either the histological type or TNM stage. Muller et al. [37] has previously reported a highly significant correlation between TFF1 expression and that of pepsinogen II, a marker of gastric differentiation, in gastric adenocarcinoma tissue. However, there was no significant relationship between TFF1 expression and the histological type of gastric cancer. Similarly, TFF1 knockout mice have been shown to develop both gastric adenomas and carcinomas [38]. Furthermore, TFF1 has been shown to be markedly down-regulated in human gastric cancer [39]. These observations may explain to a certain extent why serum TFF1 and the PG I/II ratio were not related with histological types and TNM stages in gastric cancer. With respect to TFF2, spasmolytic polypeptide (TFF2)-expressing metaplasia (SPEM) has been frequently observed in the gastric mucosa surrounding gastric cancer and TFF2 is reported to be down-regulated (83.3%) in primary gastric cancer [40]. Thus the lower level of serum TFF2 in patients with intestinal type gastric cancer may reflect the replacement of SPEM with intestinal metaplasia. In contrast, TFF3 has been reported to be up-regulated in most malignant tumors including primary gastric cancer [23-27]. Moreover, its expression has been correlated with a highly aggressive phenotype and poor prognosis [41]. Im et al. further found that TFF3 expression is higher in patients with undifferentiated type gastric cancer, and that it significantly correlated with advanced stages [39]. Thus, the results of our study are highly consistent with these reports. However, according to the histopathogenesis of gastric cancer, because TFF3 is strongly expressed by goblet cells in the normal intestine and in the intestinal metaplastic epithelium of the stomach, high expression of TFF3 would be expected in differential type and intestinal type gastric cancer. Further investigation is therefore needed to explain these mutually contradictory phenomena. Notably, other studies have recently reported that serum TFF3 is increased in patients with lung cancer, endometrial cancer, and prostate cancer, and that TFF3 is expressed in the tissue of these cancers [24,26,27]. Thus, elevated serum TFF3 levels may not be specific for gastric cancer. Therefore, the origin of high serum TFFs also needs further examination.

One limitation of this study is the biased sampling owing to its enrolment of subjects from a clinical series of hospital cases mixed with healthy people from the health check center of a hospital rather than a population-based cohort. The number of study cases was also limited. Consequently, further population-based studies or large clinical cohort studies are required to confirm the strong predictive power of serum TFF3 as well as that of its combination with the PG test, and to thereby identify the possibility of serum TFF3 as a non-endoscopic biomarker in population-based screening for gastric cancer.

## Conclusions

To evaluate their use as potential biomarkers for population-based screening, we explored the predictive power of serum TFFs compared with that of the PG test for the detection of gastric cancer. We found the firstly, the concentration of serum TFF3 may be a better biomarker of gastric cancer than the PG test. Second, combination testing of serum PG and TFF3 could improve the efficacy of gastric cancer screening. Third, the serum TFF3 level has an association with the differentiation type and TNM stage in gastric cancer. Our findings therefore support serum TFF3 concentration as biomarker for gastric cancer screening.

## Abbreviations

TFFs: Serum trefoil factors; PG: Pepsinogen; CAG: Chronic atrophic gastritis; CNAG: Chronic non-atrophic gastritis; HP: Helicobacter pylori; Ig: Immunoglobulin; ELISA: Enzyme-linked immunosorbent assay; TMB: Tetramethyl benzidine; PG +: PG positive; ROC: Receiver operating characteristic; AUC: Area under these curves.

## Competing interests

The authors declare that they have no competing interests.

## Authors’ contributions

ZH designed the study and wrote the paper. QZ and HD participated in the writing of the paper. XZ, HL, LW and DW obtained the samples and analyzed the data. All authors approved of the final manuscript prior to submission. All authors read and approved the final manuscript.

## Pre-publication history

The pre-publication history for this paper can be accessed here:

http://www.biomedcentral.com/1471-230X/14/74/prepub
